# Whole exome sequencing reveals recurrent mutations in *BRCA2* and *FAT* genes in acinar cell carcinomas of the pancreas

**DOI:** 10.1038/srep08829

**Published:** 2015-03-06

**Authors:** Toru Furukawa, Hitomi Sakamoto, Shoko Takeuchi, Mitra Ameri, Yuko Kuboki, Toshiyuki Yamamoto, Takashi Hatori, Masakazu Yamamoto, Masanori Sugiyama, Nobuyuki Ohike, Hiroshi Yamaguchi, Michio Shimizu, Noriyuki Shibata, Kyoko Shimizu, Keiko Shiratori

**Affiliations:** 1 Institute for Integrated Medical Sciences, Tokyo Women's Medical University, Tokyo, Japan; 2 Department of Surgery, Institute of Gastroenterology, Tokyo Women's Medical University, Tokyo, Japan; 3 Department of Gastroenterology, Institute of Gastroenterology, Tokyo Women's Medical University, Tokyo, Japan; 4 Department of Pathology, Tokyo Women's Medical University, Tokyo, Japan; 5 Department of Surgical Pathology, Tokyo Women's Medical University, Tokyo, Japan; 6 Department of Surgery, Kyorin University School of Medicine, Mitaka, Japan; 7 Department of Pathology, Showa University School of Medicine, Tokyo, Japan; 8 Department of Pathology, Saitama Medical University International Medical Center, Hidaka, Japan

## Abstract

Acinar cell carcinoma of the pancreas is a rare tumor with a poor prognosis. Compared to pancreatic ductal adenocarcinoma, its molecular features are poorly known. We studied a total of 11 acinar cell carcinomas, including 3 by exome and 4 by target sequencing. Exome sequencing revealed 65 nonsynonymous mutations and 22 indels with a mutation rate of 3.4 mutations/Mb per tumor, on average. By accounting for not only somatic but also germline mutations with loss of the wild-type allele, we identified recurrent mutations of *BRCA2* and *FAT* genes. *BRCA2* showed somatic or germline premature termination mutations, with loss of the wild-type allele in 3 of 7 tumors. *FAT1*, *FAT3*, and *FAT4* showed somatic or germline missense mutations in 4 of 7 tumors. The germline *FAT* mutations were with loss of the wild-type allele. Loss of BRCA2 expression was observed in 5 of 11 tumors. One patient with a *BRCA2*-mutated tumor experienced complete remission of liver metastasis following cisplatinum chemotherapy. In conclusion, acinar cell carcinomas show a distinct mutation pattern and often harbor somatic or germline mutations of *BRCA2* and *FAT* genes. This result may warrant assessment of *BRCA2* abrogation in patients with the carcinoma to determine their sensitivity to chemotherapy.

Acinar cell carcinoma of the pancreas is a rare tumor type that accounts for 1–2% of exocrine pancreatic neoplasms^1^. The prognosis for patients with acinar cell carcinoma is poor, with a 5-year survival rate of 0–72%, depending on the stage and therapy (resected or unresected)^2^,^3^,^4^. Compared with pancreatic ductal adenocarcinoma, which is far more common and well-characterized at the molecular level, the molecular features of acinar cell carcinoma have been rarely studied because of the infrequency of the disease and the lack of availability of fresh tissue suitable for large-scale molecular analysis. The lack of molecular data likely impedes development of a sensitive diagnostic marker and/or an efficient therapeutic.

Recent technological advances in DNA sequencing enable comprehensive analyses of genetic mutations in cancers. Jiao et al. have published the results of whole-exome sequencing of 23 pancreatic tumors with acinar differentiation, including 17 acinar cell carcinomas^5^. They reported an average of 64 somatic mutations per tumor (range 12–189) with enrichment of C:G-to-T:A transitions and a relatively high number of large chromosomal changes; however, no commonly mutated genes prevailed in >30% of examined samples. In the 17 acinar cell carcinomas, 4 tumors harbored somatic mutations in *SMAD4*; 3 in *JAK1, RB1*, and *TP53*; 2 in *APC, ARID1A, GNAS, MLL3, PTEN, FAT4*, and *CTNNB1*; and 1 in *BRAF, ATM, BAP1, BRCA2, PALB2, RNF43, FAT2, TSC2*, and *MSH2*. Some of the protein products of these mutated genes are druggable, which may pave the way for the development of specific drugs for acinar cell carcinoma.

We studied the molecular characteristics of 11 acinar cell carcinomas using whole-exome and target sequencing and immunohistochemical analyses (Table 1). We identified recurrent mutations of *BRCA2* and *FAT* genes, along with an unexpected contribution of germline variations, in acinar cell carcinomas.

## Results

**Figure 1:**
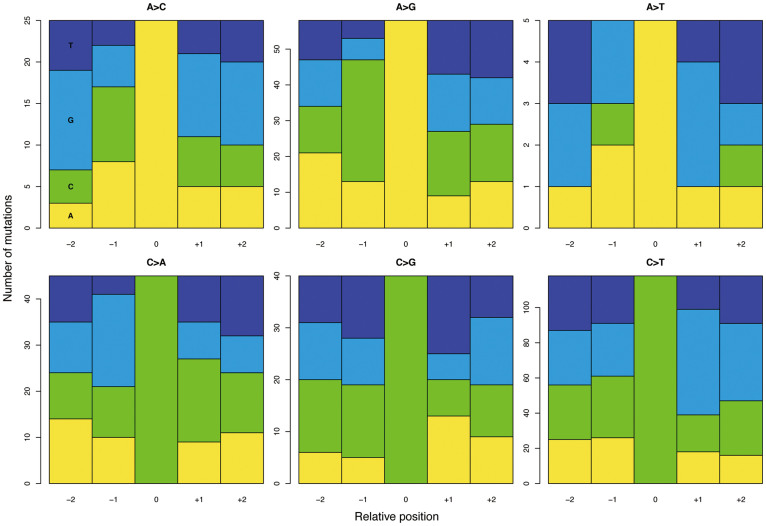
Sequence context of mutated nucleotides. Panels of nucleotide sequence context show proportions of A, C, G, and T within 2 bases before and after mutated bases. The vertical axis indicates the number of nucleotides. See Supplementary Table S2 for exact counts and proportions.

We performed whole-exome sequencing analyses of 3 acinar cell carcinomas for which frozen tissues were available using a massively parallel deep sequencer. We obtained 3 Gb of on-exon sequencing data with a mean coverage of 70 (range, 54–87) per tumor, on average. After stringent data processing to exclude false variation calls, we identified an average of 98 tumor-specific single nucleotide variations (SNVs) per tumor, including 3 nonsense variations and 62 missense variations (Table 2 and Supplementary Table S1). We also identified an average of 22 tumor-specific small insertions or deletions (indels) per tumor, including an average of 12 frameshift indels (Table 2 and Supplementary Table S1). The average mutation rate was estimated to be 3.4 mutations/Mb. We did not detect any evidence suggesting microsatellite instability that would lead to accumulation of large numbers of indels in mononucleotide repeats. We analyzed mutations for sequence context and found significant enrichment of C:G>T:A transitions (40.5%) and specific dinucleotide mutations, including CpG>TpG, CpA>CpG, and GpC>GpA (Figure 1 and Supplementary Table S2). Copy number variations calculated using the exome data revealed numerous regions of abnormalities, consistent with the results of our independent analysis using comparative genomic hybridization (Supplementary Figures S1 and S2). We also identified a number of heterozygous germline variations causing loss of the wild-type allele, which might play a role in establishing tumor phenotype (Supplementary Table S1). Because no genes showed recurrent somatic mutations, we looked for gene families harboring somatic and/or germline mutations with loss of the wild-type allele, and identified recurrent mutations of *BRCA2* and *FAT* genes. Then, we performed target sequencing analyses for the entire coding regions of *BRCA1, BRCA2, FAT1*, and *FAT4* via a semiconductor sequencer in an additional 4 cases of acinar cell carcinoma, using archival formalin-fixed and paraffin-embedded (FFPE) tissues.

We identified recurrent mutations of *BRCA2* in 3 of 7 acinar cell carcinomas in the sequencing analyses. These *BRCA2* mutations were either somatic or germline premature termination mutations accompanied by loss of the wild-type allele (Table 3 and Supplementary Figure S3). The germline mutations identified are registered in the dbSNP database, without known allelic frequencies (http://www.ncbi.nlm.nih.gov/projects/SNP) (Table 3). We also identified recurrent mutations of *FAT* genes in 4 of the 7 cases. Somatic mutations were identified in *FAT1, FAT3*, and *FAT4*, all of which were present as heterozygous with the wild-type allele. Moreover, germline mutations with loss of the wild-type allele were identified in *FAT1* and *FAT3* (Table 3 and Supplementary Figure S3). In on-line programs to predict the functional effects of human nonsynonymous SNVs, all these *FAT* mutations were predicted as at least possibly damaging by Polyphen-2^6^ (http://genetics.bwh.harvard.edu/pph2/) while only *FAT3^S2741I^* and *FAT4^E1111K^* were predicted as damaging by SIFT^7^ (http://sift.jcvi.org/) and CONDEL^8^ (http://bg.upf.edu/fannsdb/) (Supplementary TableS1). None of the *FAT1* and *FAT3* germline mutations identified here had previously been reported in public databases (dbSNPs or 1000Genomes (http://www.1000genomes.org/)). None of the somatic mutations of *BRCA2, FAT1, FAT3*, or *FAT4* identified here had previously been reported in a database of somatic mutations in cancer (COSMIC; http://cancer.sanger.ac.uk/cosmic/) or in the whole-exome data published by Jiao et al^5^.

**Figure 2:**
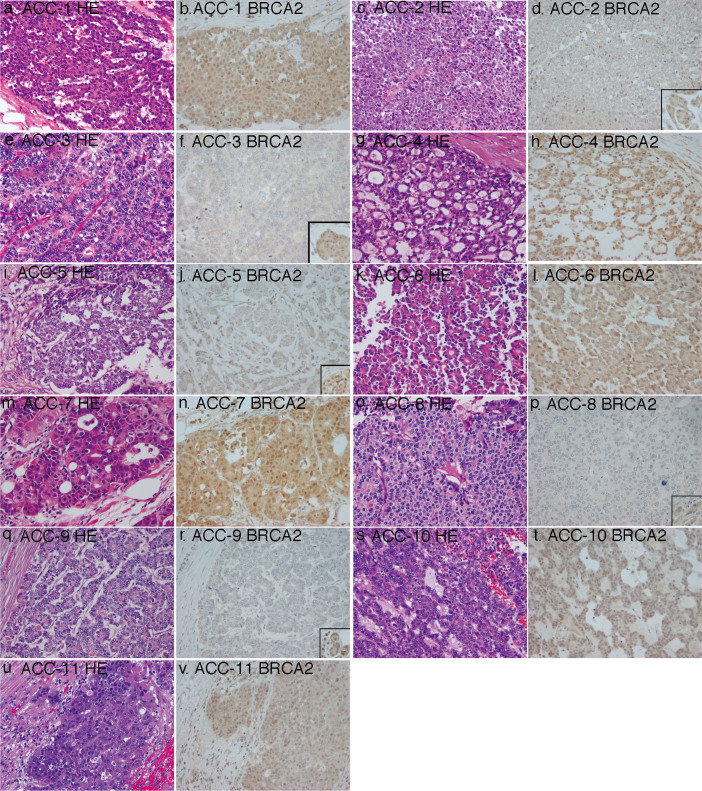
Immunohistochemical examination of the expression of BRCA2 in acinar cell carcinoma. Insets in panels d, f, j, p, and r show positive staining in normal acini in the same slide. HE, hematoxylin and eosin staining. Original magnification ×200.

The expression of the proteins encoded by these genes, namely, breast cancer type 2 susceptibility protein (BRCA2), protocadherin Fat 1 precursor (FAT1), protocadherin Fat 3 precursor (FAT3), and protocadherin Fat 4 precursor (FAT4), was examined in 11 resected acinar cell carcinomas using FFPE tissues. Five of the 11 tumors, including 3 tumors with *BRCA2* mutations, showed loss of expression of BRCA2 (Figure 2). FAT-family proteins, including FAT1, FAT3, and FAT4, were found to be expressed in all examined tumor samples, including those with mutations.

The *BRCA2* mutation is known to be associated with sensitivity to chemotherapy. In our cohort, 6 of 11 patients received chemotherapy with gemcitabine, S-1, CPT-11, and/or cisplatinum (Table 1). One of these patients, ACC-2, whose tumor harbored the somatic frameshift mutation with loss of the wild-type allele of *BRCA2*, received cisplatinum after a recurrence, with liver metastasis and experienced complete remission of the recurring tumor (Supplementary Figure S4).

## Discussion

Whole-exome sequencing allowed us to identify a characteristic pattern of genetic alterations, with numerous copy number variations, in acinar cell carcinomas. We found that acinar cell carcinomas harbored an average of 98 somatic mutations, including 65 nonsynonymous mutations, with an average frequency of 3.4 mutations/Mb per tumor. Jiao et al. have indicated that acinar cell carcinomas harbor an average of 65 nonsynonymous mutations, identical to our results^5^. Compared with other common solid cancers, the average number of nonsynonymous mutations observed per tumor in acinar cell carcinomas is greater than that observed in pancreatic ductal adenocarcinoma, 48, breast cancer, 33, glioblastoma, 35, hepatocellular carcinoma, 39, and prostate cancer, 41; comparable to that observed in gastric cancer, 53, esophageal adenocarcinoma, 57, and colon cancer, 66; and lower than that observed in melanoma, 135, and lung cancer, 147^9^. The mutation frequency of 3.4 mutations/Mb per tumor is greater than that of prostate cancer, 0.7, breast cancer, 1.2, ovarian cancer, 1.7, and endometrial cancer, 2.5; comparable to that of observed in colorectal cancer, 3.1, and diffuse large B-cell lymphoma, 3.3; and lower than that observed in lung adenocarcinoma, 8.1, and melanoma, 12.9, according to data recently published elsewhere^10^. The frequency of the C:G > T:A transition, which represented 40% of the mutations in our samples, is comparable to the finding of 35% published by Jiao et al.^5^, but slightly lower than the 53.8% frequency found in pancreatic ductal adenocarcinoma^11^. Compared to the sequence context data for the mutations found in 12 major types of cancer studied by Kandoth et al.^12^, our data also show significant enrichment of CpG>TpG and CpA>CpG. However, our finding of enrichment of GpC>GpA differs from the results published by Kandoth et al., and appears to be a unique feature of our cohort. Although the mechanism underlying this mutation pattern is unclear, C to A transversion is known to be associated with DNA injury induced by the hydroxyl radical^13^, and this result therefore indicates that acinar cell carcinoma might be associated with carcinogen-inducing hydroxyl radicals, as well as common carcinogens. Nevertheless, these data suggest that the genetic susceptibility to acinar cell carcinoma is distinct from that of pancreatic ductal adenocarcinoma, and in comparative levels to other common type of cancers.

We obtained detailed data on copy number variations by processing exome data using published methods, with some modifications for our platform^14^,^15^. We identified numerous copy number variations, including loss of 11p, 17p, and 18q, consistent with data published by Jiao et al^5^. The exome-derived detailed copy number variation data were highly consistent with data from array comparative genomic hybridization and enabled us to detect copy number variations at the individual gene level. This was particularly useful for detection of loss of the wild-type allele, which is crucial for identifying driver mutations in cancer. Using the copy number variation data, we could investigate not only somatic mutations but also germline variations with loss of the wild-type allele, which led us to discover recurrent mutations in *BRCA2* and *FAT* genes.

Recurrent premature termination mutations of *BRCA2* with loss of the wild-type allele were identified in 3 of the 7 acinar cell carcinomas analyzed by exome and target sequencing. Moreover, 5 of the 11 acinar cell carcinomas examined by immunohistochemistry revealed loss of BRCA2 expression. These results indicated that loss of functional BRCA2 may often occur in acinar cell carcinomas. *BRCA2* is a well-known susceptibility gene in hereditary breast and ovarian cancer syndrome; the encoded protein is involved in the homologous recombination pathway for double-stranded DNA break repair, which is crucial for maintenance of genome stability (information provided by RefSeq (http://www.ncbi.nlm.nih.gov/refseq/)). Consistent with our data, associations between acinar cell carcinomas and genetic aberrations of *BRCA2* have often been reported. Skoulidis reported that all 3 acinar cell carcinomas developed in patients with a germline mutation of *BRCA2*^999del5^ lacked the wild-type allele of *BRCA2*, in contrast to the observation that only 1 of 4 pancreatic ductal adenocarcinomas in patients with the same genetic phenotype lack the wild-type allele^16^. Dewald et al. reported that loss of a *BRCA2* allele was detected in 2 of 5 acinar cell carcinomas^17^. Jiao et al. reported that a somatic mutation in *BRCA2*, g.chr13:31805052C>G (referenced on hg18), p.D479E, was identified in 1 of 23 pancreatic tumors with acinar cell differentiation, although neither the allelic state of the mutation nor the state of germline variations were described for their cohort^5^. The development of acinar cell carcinoma was also reported in a patient with a *BRCA1* germline mutation^18^.

Recurrent mutations of *FAT* genes were observed in our cohort. Human *FAT* genes include *FAT1, FAT2, FAT3*, and *FAT4*, and each of these genes encodes a similar large transmembrane protein consisting of multiple extracellular cadherin domains and a cytoplasmic domain that can interact with signaling molecules, which is homologous to *fat* in *Drosophila*, which has a tumor suppressor function^19^,^20^. *FAT* genes have been shown to harbor somatic mutations in cancers of several organs, including pancreatic ductal adenocarcinoma^21^,^22^. According to COSMIC, 0.6% (1/167) of pancreatic ductal adenocarcinomas harbor mutations in *FAT1*, 2.1% (4/189) in *FAT2*, 0.5% (1/189) in *FAT3*, and 0% (0/189) in *FAT4*. Acinar cell carcinomas are also reported to harbor nonsynonymous somatic mutations of *FAT2* and *FAT4* at respective rates of 5.9% (2/17) and 11.76% (2/17); however, no mutations in *FAT1* or *FAT3* are reported in COSMIC. In the present study, we identified nonsynonymous somatic missense mutations in *FAT1*, *FAT3*, and *FAT4*. These missense mutations did not appear to affect protein expression, because our immunohistochemical results showed no alteration of expression. Moreover, we identified germline missense mutations in *FAT1* and *FAT3* resulting in loss of the wild-type allele, which suggests that these germline mutations are likely to be selected and to contribute to the phenotype of acinar cell carcinoma. To the best of our knowledge, this is the first study to report the selection of germline mutations of *FAT* genes and their apparent contribution to a phenotype of human cancer. Although Jiao et al. did not examine germline variations, they identified somatic mutations of *FAT2*, p.R2678Q, and *FAT4*, p.H3160Q and p.I64M, all involving the cadherin domains, in acinar cell carcinomas^5^.

BRCA2 is involved in the repair of double-stranded breaks in DNA. Therefore, cancer cells lacking functional BRCA2 are vulnerable to drugs that induce double-stranded DNA breaks, e.g., mitomycin-C, cisplatinum, and poly ADP-ribose polymerase inhibitor^23^,^24^. In our cohort, one patient, ACC-2, with a tumor harboring the loss-of-function mutation in *BRCA2* developed multiple liver metastases but then experienced complete remission after receiving cisplatinum. Although this was the only patient treated with cisplatinum in our cohort, this observation indicated that an acinar cell carcinoma with a loss-of-function mutation in *BRCA2* may respond well to chemotherapy with cisplatinum, which indicates the particular importance of *BRCA2* status as a biomarker for sensitivity to chemotherapy in acinar cell carcinomas.

Unlike Jiao, et al.^5^, we did not observe somatic mutations in *SMAD4*, *JAK1, RB1*, *TP53*, *APC, ARID1A, GNAS, MLL3, PTEN, CTNNB1*, *BRAF, ATM, BAP1, PALB2, RNF43, TSC2*, and *MSH2* in our 3 cases exmained by whole-exome sequencing.

In conclusion, whole-exome sequencing revealed that acinar cell carcinomas harbored an average of 98 somatic mutations per tumor, including 65 nonsynonymous mutations, with an average frequency of 3.4 mutations/Mb. The observed mutation frequency exceeds that of pancreatic ductal adenocarcinoma, but is comparable to that of digestive tract cancers. Acinar cell carcinoma may commonly harbor somatic or germline loss-of-function mutations of *BRCA2* and *FAT* genes. This result may warrant screening for *BRCA2* abrogation to assess sensitivity to chemotherapy in patients with acinar cell carcinoma.

## Methods

### Materials

Eleven surgically resected cases of acinar cell carcinoma from the authors' institutions were studied (Table 1). All cases were histopathologically reviewed, and the diagnosis was confirmed by immunohistochemical demonstration of trypsin expression in tumor cells. Of these, 3 recent cases for which frozen tissues were available were subjected to whole-exome sequencing analysis. Four additional cases for which archival FFPE tissues were available were used for target semiconductor sequencing analyses. FFPE tissues for all 11 cases were subjected to immunohistochemical analysis. The methods were performed in accordance with the relevant guidelines and regulations. This study was approved by the ethical committee of Tokyo Women's Medical University. Relevant informed consent was obtained from subjects.

### Whole-exome sequencing

Tumor and normal tissues were dissected and collected separately from frozen sections under microscopic guidance. DNA was extracted using a ChargeSwitch® gDNA Mini Tissue Kit (Life Technologies, Carlsbad, CA). The extracted DNA was constructed into a fragment library using a SOLiD Fragment Library Construction Kit (Life Technologies) or the AB Library Builder System (Life Technologies). Constructed libraries were subjected to whole-exome enrichment using a SureSelect Human All Exon Kit (Agilent Technologies Inc., Santa Clara, CA) or a TargetSeq™ Target Enrichment Kit (Life Technologies). The prepared exome libraries were sequenced using the massively parallel deep sequencer SOLiD 4 or 5500xl SOLiD System (Life Technologies) using the paired-end sequencing method. Data were analyzed using LifeScope software (Life Technologies) with mapping on the Human Genome Reference, GRCh37/hg19 (The Genome Reference Consortium; http://www.ncbi.nlm.nih.gov/projects/genome/assembly/grc/index.shtml). All procedures were performed according to the manufacturers' instructions. Obtained data were annotated and stringently filtered to exclude false variation calls using our previously described programs developed in-house^25^.

### Copy number variation using whole-exome sequencing data

We calculated copy number variations for each exon from the exome sequencing data using our in-house program with guidance from the published literature^14^ and ExomeCNV^15^ using the R platform (http://www.r-project.org/).

### Array comparative genomic hybridization

Extracted genomic DNA was used as a template. Genomic copy number aberrations were analyzed using the SurePrint G3 Hmn CGH 60 k Oligo Microarray (Agilent Technologies, Santa Clara, CA) according to previous reports^26^. Briefly, 250 ng each of target and reference DNA was digested with the restriction enzymes, AluI and RsaI. Cy-5 (target) or Cy-3 dUTP (reference) were incorporated using the Klenow fragment. The array was hybridized with labeled DNAs in the presence of Cot-1 DNA (Life Technologies) and blocking agents (Agilent Technologies) for 24 h at 65°C, washed, and scanned on the scanner (Agilent Technologies). Data were extracted using Agilent Feature Extraction software ver. 10 with the default settings for aCGH analysis. Statistically significant aberrations were determined using the ADM-II algorithm in Agilent Genomic Workbench software version 6.5 (Agilent Technologies). Genomic locations were referenced according to Genome Reference Consortium GRCh37/hg19.

### Semiconductor sequencing

Semiconductor sequencing using an IonTorrent Sequencer (Life Technologies) was performed to detect mutations in the entire coding exons of *BRCA1, BRCA2, FAT1*, and *FAT4* in FFPE tissues from archival cases of acinar cell carcinoma, according to the manufacturer's instructions. Tumor and normal tissues were dissected and collected separately under microscopic guidance. DNA was extracted using a QIAamp DNA FFPE Tissue Kit (Qiagen GmbH, Hilden, Germany) according to the manufacturer's instructions. The semiconductor sequencing analysis was performed with a custom designed Ion AmpliSeq^TM^ Primer Pool, an Ion AmpliSeq^TM^ Library Kit, Ion Xpress^TM^ Library Barcode Adaptors, Ion One Touch^TM^ 2, and an Ion PGM^TM^ sequencer, according to the manufacturer's instructions (Life Technologies).

### Sanger sequencing

For validation of the whole-exome and the semiconductor sequencing data, DNA was amplified by polymerase chain reaction (PCR) using the primers listed in Supplementary Table S3 and the AccuPrime PCR system (Life Technologies). The amplified products were treated with ExoSAP-IT (GE Healthcare, Chalfont St. Giles, Buckinghamshire, UK) and sequenced using BigDye Terminator and a 3130xl Genetic Analyzer (Life Technologies) according to the manufacturers' instructions. We assessed loss of the wild-type allele by confirming suppression of electropherograms corresponding to nucleotide sequences of the wild-type allele.

### Immunohistochemistry

Indirect immunohistochemical staining of paraffin-embedded tissues using the streptavidin and biotin system was performed using a Histofine SAB-PO kit (Nichirei Biosciences Inc., Tokyo, Japan) as described previously^27^. The antibodies used were a rabbit polyclonal anti-BRCA2, #CA1033, produced by using a carboxyl-terminal region (amino acids 3245–3418) of human BRCA2 as an immunogen (Merck Millipore, Billerica, MA), a rabbit polyclonal anti-FAT1 (Sigma, St. Lois, MO), a rabbit polyclonal anti-FAT3 (Atlas Antibodies, Stockholm, Sweden), a rabbit polyclonal anti-FAT4 (Novus Biologicals, Littleton, CO), and a rabbit polyclonal anti-human trypsin (Meridian Life Sciences Inc., Memphis, TN). Antigen retrieval and dilution of antibodies were performed according to the manufacturers' instructions. Staining of tumor tissue was evaluated by comparing with staining of normal tissues.

### Statistics

The sequence context of mutated nucleotides was examined by the exact test for goodness of fit in order to determine whether or not nucleotides before or after mutations were random, with a probability of 0.25 for each nucleotide. Statistical analysis was performed using the R platform. *P*-values < 0.05 were considered statistically significant.

## Author Contributions

T.F. designed the study and wrote the manuscript. T.F., H.S., S.T., M.A., Y.K. and T.Y. prepared and performed experiments. T.F., Y.K., T.H., M.Y., M.Su., N.O., H.Y., M.Sh., N.S., K.Shim. and K.Shir. obtained and analyzed clinicopathological data. All authors reviewed the manuscript.

## Supplementary Material

Supplementary Information Supplementary Table S1

Supplementary Information Supplementary Data

## Figures and Tables

**Table 1:** Clinicopathological information of studied cases

Sample	Age	Sex	Site	Size (cm)	Chemotherapy	Survival (months)	Recurrence	Outcome	Analysis
ACC-1	78	M	BT	4	GEM, S-1	16	Liver	DOD	WE, IHC
ACC-2	67	M	B	5.5	CDDP	59	Liver (CR)	Alive	WE, IHC
ACC-3	78	F	T	8.5	None	21	None	Alive	WE, IHC
ACC-4	68	M	BT	12.3	None	15.7	Liver	DOD	TS, IHC
ACC-5	59	M	B	4.7	LAK, DV, S-1	23	Liver, Peritoneum	DOD	TS, IHC
ACC-6	56	F	H	5	None	101	None	Alive	TS, IHC
ACC-7	71	M	H	2	GEM, S-1, CPT-11	12	Liver	DOD	TS, IHC
ACC-8	53	M	T	4.3	GEM	23	Liver	DOD	IHC
ACC-9	58	M	BT	18	None	94	None	Alive	IHC
ACC-10	55	M	H	7	None	159	None	Alive	IHC
ACC-11	70	M	BT	10	S-1	6	Liver	DOD	IHC

Abbreviations are B, pancreatic body; BT, pancreatic body and tail; CDDP, cisplatinum; CR, complete remission; DOD, dead on disease; DV, dentritic cell vaccination; F, female; GEM, gemcitabin; H, pancreatic head; IHC, immunohistochemistry; LAK, lymphokine-activated killer cell; M, male; T, pancreatic tai; TS, target sequencing; and WE, whole-exome sequencing.

**Table 2:** Number of somatic variations obtained by whole exome analysis

Sample	Somatic mutations	Nonsynonymous mutations	Nonsense mutations	Missense mutations	Indels	Frameshift indels	Mutation rate (/Mb)
ACC-1	108	72	1	71	22	13	3.9
ACC-2	78	52	3	49	16	12	2.9
ACC-3	107	72	5	67	28	12	3.5
Average	97.7	65.3	3	62.3	22	12.3	3.4

Abbreviations are Indels, insertions or deletions; and Mb, megabases.

**Table 3:** Mutations in *BRCA2* and *FAT* genes in acinar cell carcinomas

Gene	Mutation	Wild type allele	Status	dbSNP	Sample	Expression
*BRCA2*	c.8297delC: p.T2766NfsX10	Loss	Somatic	-	ACC-2	Loss
*BRCA2*	c.7115C>G: p.S2372X	Loss	Germline	rs80358943	ACC-3	Loss
*BRCA2*	c.4021delT; p.S1341QfsX33	Loss	Germline	rs397507702	ACC-5	Loss
*FAT1*	c.9046G>A: p.A3016T	Loss	Germline	-	ACC-2	Retain
*FAT1*	c.11216C>T; p.A3739V	Retain	Somatic	rs74511500	ACC-4	Retain
*FAT3*	c.6449C>T: p.S2150F	Loss	Germline	-	ACC-3	Retain
*FAT3*	c.8222G>T: p.S2741I	Retain	Somatic	-	ACC-3	Retain
*FAT4*	c.3331G>A: p.E1111K	Retain	Somatic	-	ACC-1	Retain
