# Supplementary material for: Whole exome sequencing reveals recurrent mutations in BRCA2 and FAT genes in acinar cell carcinomas of the pancreas
Source: Sci Rep. 2015 Mar 6;5:8829. doi: 10.1038/srep08829 (PMC4351513; doi:10.1038/srep08829)
Supplement: Supplementary Information — Supplementary Data [file srep08829-s2.pdf]

Whole exome sequencing reveals recurrent mutations in *BRCA2* and *FAT* genes in acinar cell carcinomas of the pancreas

Toru Furukawa[1, 5*], Hitomi Sakamoto[1, 6], Shoko Takeuchi[1], Mitra Ameri[1], Yuko Kuboki[1, 3], Toshiyuki Yamamoto[1], Takashi Hatori[2], Masakazu Yamamoto[2], Masanori Sugiyama[6], Nobuyuki Ohike[7], Hiroshi Yamaguchi[8], Michio Shimizu[8], Noriyuki Shibata[4], Kyoko Shimizu[3], and Keiko Shiratori[3].

[1]Institute for Integrated Medical Sciences, [2]Department of Surgery and [3]Department of Gastroenterology, Institute of Gastroenterology, [4]Department of Pathology, [5]Department of Surgical Pathology, Tokyo Women's Medical University, Tokyo, Japan; [6]Department of Surgery, Kyorin University School of Medicine, Mitaka, Japan; [7]Department of Pathology, Showa University School of Medicine, Tokyo, Japan; and [8]Department of Pathology, Saitama Medical University International Medical Center, Hidaka, Japan

***Correspondence to:**

Toru Furukawa

Institute for Integrated Medical Sciences, Tokyo Women's Medical University

8-1 Kawadacho, Shinjuku, Tokyo, 162-8666 Japan

Tel.: +81-3-3353-8112 ext. 29675

Fax: +81-3-5269-7667

E-mail: furukawa.toru@twmu.ac.jp

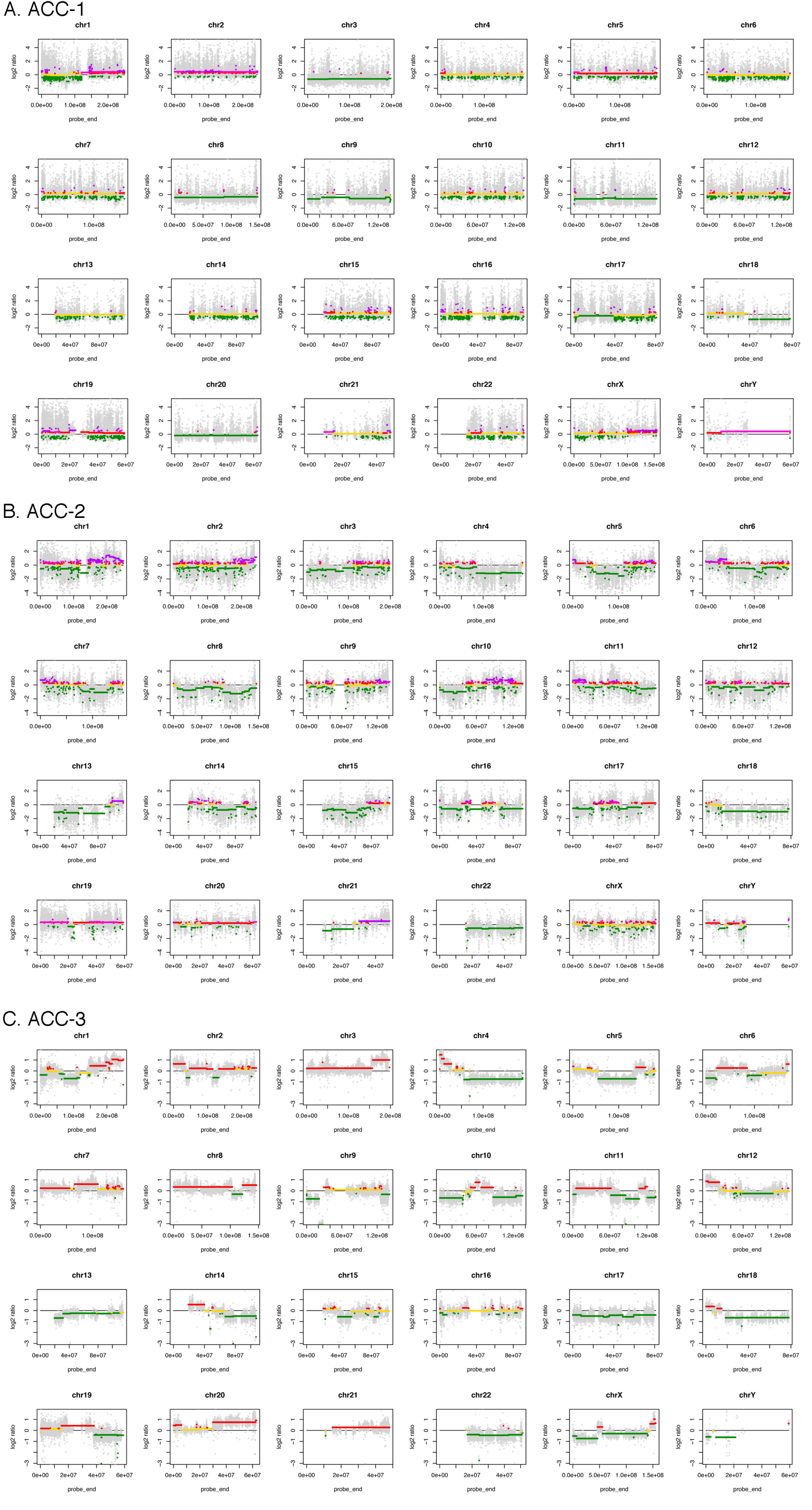

Supplementary Figure S1. Plots of copy number variations calculated from exome data

A. ACC-1

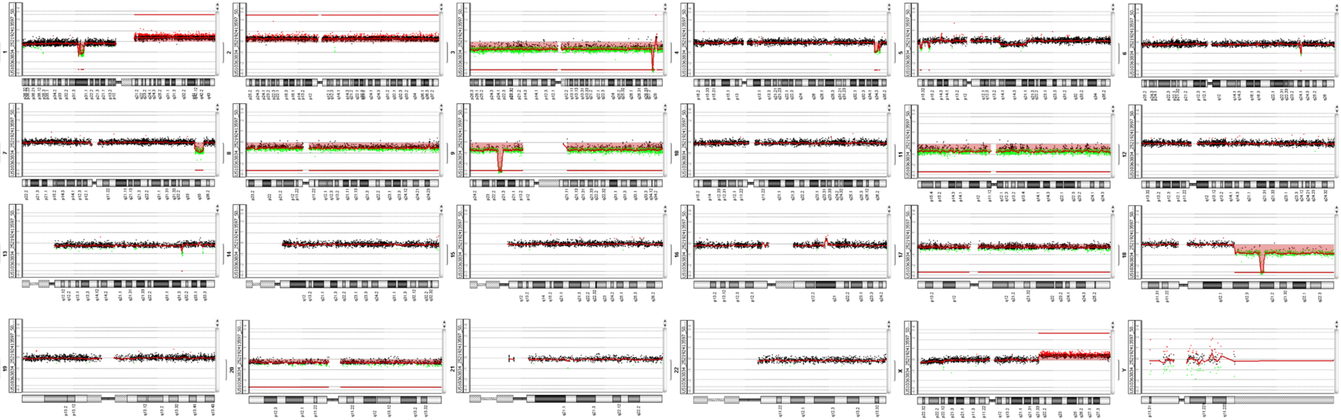

B. ACC-2

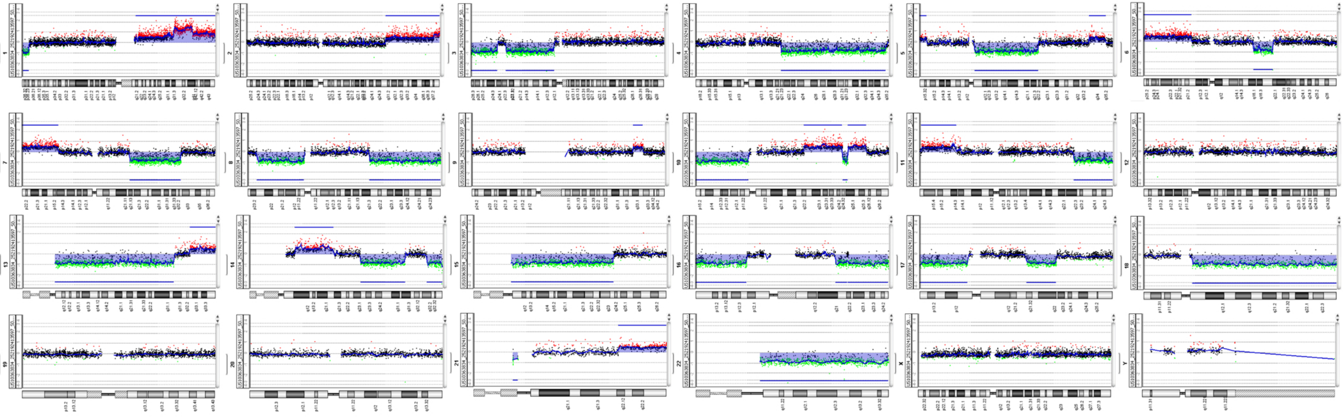

Supplementary Figure S2. Plots of the comparative genomic array hybridization.

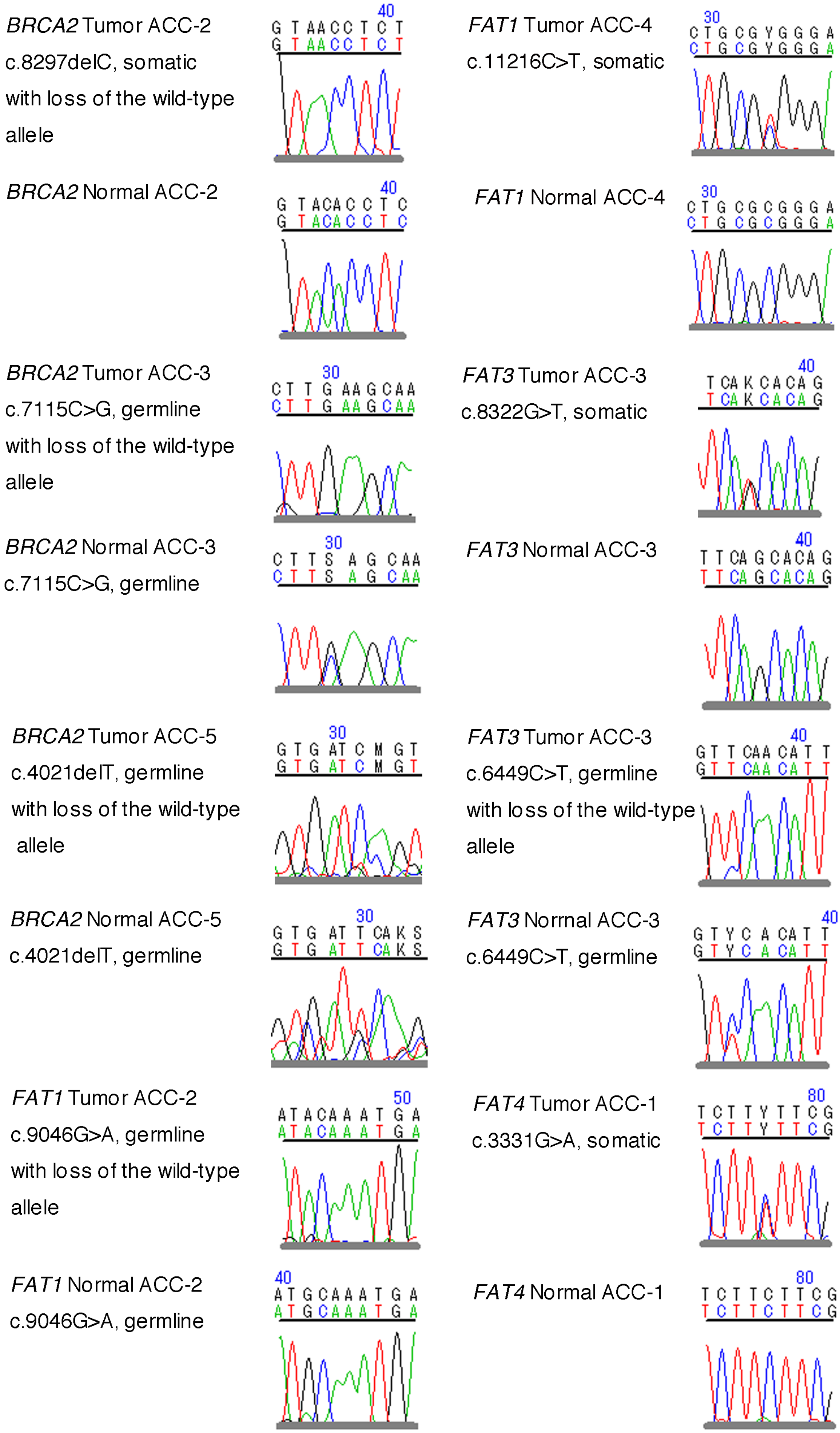

Supplementary Figure S3. Mutations validated by Sanger sequencing.

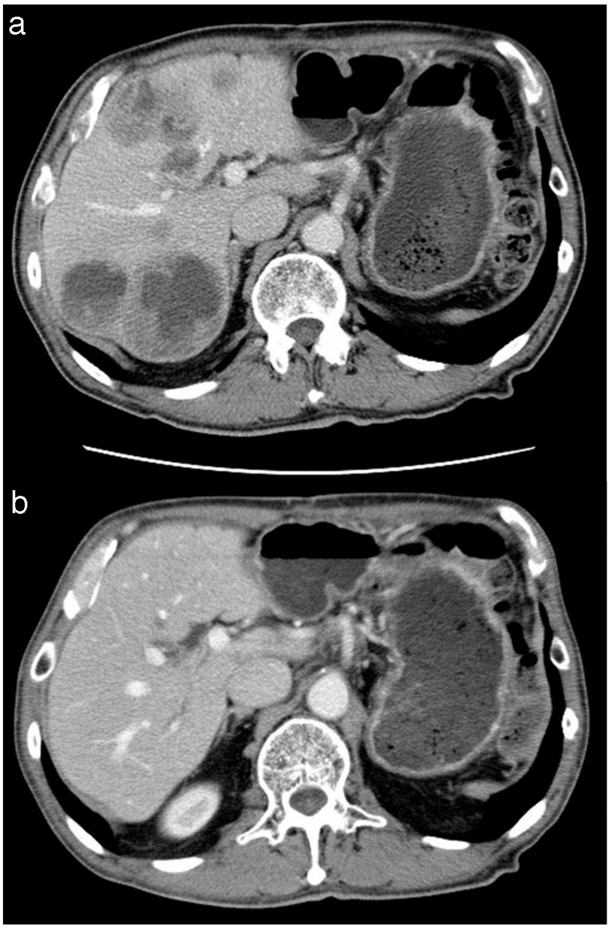

Supplementary Figure S4. Computed tomography of patient ACC-2 with acinar cell carcinoma and a somatic frameshift deletion accompanied by the loss of the wild type of BRCA2. The patient developed liver metastasis despite having gemcitabine and S-1 treatment (panel a). Subsequently, the patient underwent chemotherapy with S-1 and cisplatinum, and the metastatic tumor vanished (panel b). The patient has been recurrence-free for 5 years.

Supplementary Table S2. Sequence context of mutated bases

Mutation					A>C				
Position	-2		-1		0	+1		+2	
	Count	Ratio	Count	Ratio	Count	Count	Ratio	Count	Ratio
A	3	0.12	8	0.32	25	5	0.2	5	0.2
C	4	0.16	9	0.36	0	6	0.24	5	0.2
G	12	0.48	5	0.2	0	10	0.4	10	0.4
T	6	0.24	3	0.12	0	4	0.16	5	0.2
Total	25	1	25	1	25	25	1	25	1
Exact Test P value	-			0.31760054			0.38115794	-	

Mutation					A>G				
Position	-2		-1		0	+1		+2	
	Count	Ratio	Count	Ratio	Count	Count	Ratio	Count	Ratio
A	21	0.362069	13	0.22413793	58	9	0.15517241	13	0.224138
C	13	0.224138	34	0.5862069	0	18	0.31034483	16	0.275862
G	13	0.224138	6	0.10344828	0	16	0.27586207	13	0.224138
T	11	0.189655	5	0.0862069	0	15	0.25862069	16	0.275862
Total	58	1	58	1	58	58	1	58	1
Exact Test P value	-			7.69E-08			0.38900691	-	

Mutation					A>T				
Position	-2		-1		0	+1		+2	
	Count	Ratio	Count	Ratio	Count	Count	Ratio	Count	Ratio
A	1	0.2	2	0.4	5	1	0.2	1	0.2
C	0	0	1	0.2	0	0	0	1	0.2
G	2	0.4	2	0.4	0	3	0.6	1	0.2
T	2	0.4	0	0	0	1	0.2	2	0.4
Total	5	1	5	1	5	5	1	5	1
Exact Test P value	-			0.765625			0.4140625	-	

Mutation					C>A				
Position	-2		-1		0	+1		+2	
	Count	Ratio	Count	Ratio	Count	Count	Ratio	Count	Ratio
A	14	0.311111	10	0.22222222	0	9	0.2	11	0.244444
C	10	0.222222	11	0.24444444	45	18	0.4	13	0.288889
G	11	0.244444	20	0.44444444	0	8	0.17777778	8	0.177778
T	10	0.222222	4	0.08888889	0	10	0.22222222	13	0.288889
Total	45	1	45	1	45	45	1	45	1
Exact Test P value	-			0.0087781			0.1424529	-	

Mutation					C>G				
Position	-2		-1		0	+1		+2	
	Count	Ratio	Count	Ratio	Count	Count	Ratio	Count	Ratio
A	6	0.15	5	0.125	0	13	0.325	9	0.225
C	14	0.35	14	0.35	40	7	0.175	10	0.25
G	11	0.275	9	0.225	0	5	0.125	13	0.325
T	9	0.225	12	0.3	0	15	0.375	8	0.2
Total	40	1	40	1	40	40	1	40	1
Exact Test P value	-			0.07855316			0.07933823	-	

Mutation					C>T				
Position	-2		-1		0	+1		+2	
	Count	Ratio	Count	Ratio	Count	Count	Ratio	Count	Ratio
A	25	0.211864	26	0.22033898	0	18	0.15254237	16	0.135593
C	31	0.262712	35	0.29661017	118	21	0.1779661	31	0.262712
G	31	0.262712	30	0.25423729	0	60	0.50847458	44	0.372881
T	31	0.262712	27	0.22881356	0	19	0.16101695	27	0.228814
Total	118	1	118	1	118	118	1	118	1
Exact Test P value	-			0.65970525			8.51E-09	-	

Supplementary Table S3. Primers used for validation by Sanger sequencing

Primer	sequence
HSGP2_E24_G1	GTCCCAGGTGACCTCCTATG
HSGP2_E24_G2	GCTCCTGGGCCACATGGTGC
HSGP2_E56_G1	GTGTTGGGCAGCTCCGTGCC
HSGP2_E56_G2	TTGCAAGAGTGGGGGGCCTC
BAI2_E23_G1	CTGGGTGTCTGACACCCTTC
BAI2_E23_G2	CCCTTCCAAGATGCTGAACG
BAI2_E26_G1	TGCTGTCTTCAACTCCGCGC
BAI2_E26_G2	AGAAGCCAAGGGCCTGGCAG
MMEL1_E20_G1	GCAGCCCTCCTCTGCCCCTG
MMEL1_E20_G2	GGTCCCAGGAGTAGTTGCCG
MMEL1_E22_G1	AAGGACCAGCAGCTGCCCGG
MMEL1_E22_G2	GGCCCTCCAGAGATGAGTGG
C1orf216_Ex2_G1	CCTCTGACAACCAAGCCTTC
C1orf216_Ex2_G2	GGAGCAGACTGTGCCAGCAC
THRAP3_E11_G1	GCTGGGGCAGAGGCAACTAC
THRAP3_E11_G2	GTTATCCCCAGACACATACC
MACF1_E53_G1	ACAGGCCCTGGAGATGTCTC
MACF1_E53_G2	GTTCCTGGTACTGGGTGCTC
MACF1_E58_G1	GACTTTTCTTCCTGAGCAGC
MACF1_E58_G2	CAGGTTCTGAGTTAGCAAGC
TTLL7_E19_G1	ATATCGGATTTTCAACCGGG
TTLL7_E19_G2	CAAGCATTTTATTAGGATATCTC
TTLL7_E14_G1	TTGTGGAGATTAATCAACTTGG
TTLL7_E14_G2	CTTGGTAGTTTTTCCCATCAAC
SRRM1_E6_G1	CTCCTCTCTTCCAATCCATG
SRRM1_E6_G2	GAGATCGAGAATGACTGCGC
ELTD1_E14_G1	GGGAGATATGTTGACTATACG
ELTD1_E14_G2	GATGCGTGCACAACATGGAG
ELTD1_E6_G1	GGGATACATTTGTAGTTTGGG
ELTD1_E6_G2	CAAACTCTGTGGTCTTTTGG
LGR6_E18_G1	GGTCTTAGACCCCAAAGATG
LGR6_E18_G2	ACTGCCCAGAGGCCTTGAAG
LGR6_E20_G1	TGGCCTTCCTCAGCTTTGCC
LGR6_E20_G2	CGAAGGTCATCCCGGAAGTG
GMPPB_E8b_G1	GGCCCACTTGTATTTTTCCC
GMPPB_E8b_G2	GGCATCCCGCAGCACCGTGC
GMPPB_E3_G1	ATCCTGGTAGAACCTGACTG
GMPPB_E3_G2	CTTTTCCTGGCCTTAAACCC
FRG1_E6_G1	AAACACTTAATGTTTCTCCC
FRG1_E6_G2	TTGATCATTTCTTCTTCTCC
FRG1_E6_G3	GTCTAAACACTTAATGTTTCTCCC
FRG1_E6_G4	ACCTTGATCATTTCTTCTTCTCC
DENND4B_E15_G1	CAGAGCTACGGGCTGAGTTG
DENND4B_E15_G2	TCAGTGCGGTCCACCCACTG
DENND4B_E18_G1	TCCTGGGGGCTGCTCAGTTC
DENND4B_E18_G2	CCCCAAGCCTGTCTCACCAG
PTPRC_E11_G1	AGAAAACCTTGAACCCGAAC
PTPRC_E11_G2	AAGAACATGAAGAGAGAAGC
PTPRC_E27_G1	CCTTCATATAGGAGCTGGAG
PTPRC_E27_G2	TATATGACTGTGTGTCCCAC
ADD3_E11_G1	CTCATCTAAAGTTAGTGGTGG
ADD3_E11_G2	GTCTCCCCTGGCTATAGAAG
ADD3_E14_G1	GTCCTGTCACCTGAAGGCTC
ADD3_E14_G2	ATCCCCCAATCTGATCTACC
PLEKHH2_E2_G1	CCAGTAGATTGGAAGGAACG
PLEKHH2_E2_G2	GCAGACCCTAAATAGCTGAC
PLEKHH2_E22_G1	GAGAAGCAAGACCCTCAAGG
PLEKHH2_E22_G2	TCTGTTCCCTATAACAGAGG
INCENP_E19_G1	CAAGCCCCGCTATCACAAGC
INCENP_E19_G2	AAGAAGGCCGCAGGCCAGCC
ARAP1_E30_G1	GTGAGGACCGCAGCCTCCTG
ARAP1_E30_G2	GTGTACAGGTGCCCACCCTG
SYNE2_E47_G1	TATTGACTGATCTGTGTGAC
SYNE2_E47_G2	TATATTGTGGAGAACCAAGG
SYNE2_E99_G1	GAGAAGAAATGAGTTTGGGC
SYNE2_E99_G2	ATTTGTTGCTGGCCTTGATC
MSLN_E6_G1	CTGGGTGGACATTGCAGGGG
MSLN_E6_G2	GTGCCAAGGCCACAGCCAGC
MSLN_E14a_G1	CACGGCCTGAGGTTATGCTG
MSLN_E14a_G2	GGTGTCTTTGTCTAGCTGGC
JUP_E5_G1	CGTACTAACCCCTGCCCACC
JUP_E5_G2	TTCAGGCCTCGGGAGAGTTG
POLRMT_E5_G1	TTCACCGCCGTTCTGCTGTC
POLRMT_E5_G2	AGTCTGCCGGGGCCCACGTG
POLRMT_E15_G1	AAGCAAGTGGACGGGATCCC
POLRMT_E15_G2	TAGGGCTGGATGACGGGGAC
ZNF208_E4_G1	TACTGGAAAGAAACATTTGC
ZNF208_E4_G2	TAGTAAGGATTGCAGATTGG
ZNF587_E3_G1	GCTTGTTCACACTGGAGAAG
ZNF587_E3_G2	CAGTATGACCTCGCTGATGG
ZNF417_E3_G1	AGCCTTATTAGCCATCAGCG
ZNF417_E3_G2	AGATTTCCCACATTCCCCAC
OTOF_E44_G1	GAGCTGACACTGAGGTTGCC
OTOF_E44_G2	CCTACTGCCCGAGCAGGAAG
TTN_E35_G1	GCAATGATCTTGTTCAGGAC
TTN_E35_G2	ATCCACTGGACACCTTTGAC
TTN_E149_G1	TGGAAACTGGAAACGTGGTC
TTN_E149_G2	AGTTATCTACCTTCAACTGG
SPEG_E32_G1	TCAGCTACAAATGCCACCTG
SPEG_E32_G2	TTCAGAATCCGAGGAGGATG
SPEG_E33_G1	GCTCCGCTGGGGCTTCTCTC
SPEG_E33_G2	AGGGTCCTCACCCAGCAGGC
GIGYF2_E25_G1	TCCTTCTGGAAAGGAAGAGG
GIGYF2_E25_G2	TCCGTTCTTCTTCCTCATGC
GIGYF2_E30_G1	AAGCAGTTCCTTGAGCGCCG

GIGYF2_E30_G2	TACCGCATACACCACACTAC
ULK4_E7_G1	GGTGATAGCAGACTTCTTTC
ULK4_E7_G2	ACGTTGGATAAAGGTACACC
ULK4_E13_G1	TCTTCCTCAGCATCTTACTG
ULK4_E13_G2	CAACTCAGTTGTTAGAGTGT
ULK4_E20_G1	AGGACCCATTTTGTGGTACC
ULK4_E20_G2	CTTGCTGTTTAAAGATACCC
BSN_E5_G1	CAAACACCACATCGACCCAG
BSN_E5_G2	TGGAGGCACTGGCATGTACC
KCNIP4_codon128_G1	GGGTTGTAGCCCTTACTTAC
KCNIP4_codon128_G2	ACCTCTTTAGTGATGTAGCC
CDKL3_E6_G1	ATATCTTTTCCAAGAGCCCC
CDKL3_E6_G2	CATACATGAACTATATCTGCC
TTBK1_E2_G1	GACACCCCTCCCTCTGGCTG
TTBK1_E2_G2	TCTGTCCCGCCGGGTCACTC
TTBK1_E14_G1	TCCTCAAGTCTGAGCCCAAG
TTBK1_E14_G2	GGTGGGTTTTCTCCGCAATG
PRKDC_E19_G1	GAGTCTGAAACACTCTCCTG
PRKDC_E19_G2	ACCGCACCCAGCCCCAATTC
PRKDC_E50_G1	TTTCCAGAATGGAATGCCGC
PRKDC_E50_G2	GTTGTGTCTAAACACAGCTC
ZNF674_E6a_G1	CAACTCTCATTGTGCATCAC
ZNF674_E6a_G2	TCCCACTCTCAAGTATAATC
ZNF674_E6a_G3	TAGAAGTCTGGGAAGTTGAC
ZNF674_E6a_G4	TGATGTTTTGAACACCTTTC
SPEF2_E4_G1	AGACTTAGACACATGATACC
SPEF2_E4_G2	TTCTCAAAATGCAAATGGGC
TCF3_E19_G1	TCTCCACAACCCAGCCCTGC
TCF3_E19_G2	CGTTATTGGCCATGCGCCTC
PPEF2_E13_G1	TGAAGTTGGCAGCAACAGAG
PPEF2_E13_G2	GCTGACATAGCCAAATTCTG
RNF216_E4a_G1_	CCCAATCTCATCAAACCAGC
RNF216_E4a_G2	CCCCAGAATCAAACAATGGG
CTNND2_E20_G1	CGCTGTGGGATGAGATGCAG
CTNND2_E20_G2	TTTAGCTCCGTGGTAGGTGG
TNR_E15_G1	ACAGAAGCCCTGCTGCAGTG
TNR_E15_G2	AGCTCCAATTCCCACCATTC
PAPPA_E4_G1	TGAGATTGGTCACAGCCTGG
PAPPA_E4_G2	GTATCATTGCAGAGGTCTCC
TOP1_E4_G1	CCAAACACAAAGATGGAAGC
TOP1_E4_G2	ACCAAGGGATCTTACTATGC
IL1RL1_E11_G1	ATGTTGTCTACCCACGGAAC
IL1RL1_E11_G2	CCAGGTAGCATATCTCTCCC
GRK7_E4_G1	ACCCTTCAGTGGTTTATGCC
GRK7_E4_G2	TCTTCCTGCCATGCTATAGG
LAMC1_E14_G1	ATCGATCCCAATGCAGTTGG
LAMC1_E14_G2	GACAGAATCTGATCAAAGGC
SPAG5_E18_G1	GCTTCAGAGTCTTTGTTCCC
SPAG5_E18_G2	TTGGACCCTTGGCATAAGAG
MTOR_E31_G1	CAACTCCACCAGCAGTGCTG
MTOR_E31_G2	CATCCTTCACAGGGTGCCTG
AFG3L2_E16_G1	CATGAATGAAAAGGTTGGGC
AFG3L2 E16 G2	TGTGAGAAGAGCTACTGTTC
ZMYND12_E4_G1	ATCTATTCCAAGCCCAGTGG
ZMYND12_E4_G2	AGATGATAACGGGCCTCTTC
GLT8D2_E7_G1	CTCTCCCCCACCATCATTTC
GLT8D2_E7_G2	TTCTTGGCAGTTAGCGAGAG
NFKB1_E8_G1	AGATGGACCTCAGCGTGGTG
NFKB1_E8_G2	TGTGCCCCCTGTTGGAAGTG
STRADB_E12b_G1	CAACAGCCTTATTTTGAGTTTC
STRADB_E12b_G2	GGCTCAGTCCAAGGTAACAC
FAT4_E1_G1	AACCCCTTAGTGCTACTGTG
FAT4_E1_G2	TCTCCATTTGGCCCAAAGTC
ZNF2_E5_G1	AAAGCCTTTAGCCAGCGGTG
ZNF2_E5_G2	GCGTAACGCCGTTGATGTTG
DNAH3_E62_G1	GAGCGCAATGTTTCTGCATC
DNAH3_E62_G2	GGCATGTCTGTTGGAAGCTC
TRPV3_E10_G1	TAGAAAACGGTCCCTCCCTC
TRPV3_E10_G2	GCTTGGCAAACTTCTTCCAC
RASAL3_E11_G1	TATCTGGCTGTGCTGGGCTG
RASAL3_E11_G2	AGACCTCCTCGCAGCTGTTC
DGK1_E10_G1	ATCCTAACAGTGCTGATTGC
DGK1_E10_G2	GTGAGAGTTGTGTCGTCAAG
PCDH11X_E5a_G1	TGGCACCATAACTGTCGTTG
PCDH11X_E5a_G2	TCATCTGCCTGTTTTCTGGG
PDE6B_E20_G1	TTCTCGTGGCTGCTGAGTTC
PDE6B_E20_G2	AAGAGGGGTCTCTAACACCC
FUT2_E2_G1	GCTACAGCTCCCTCATCTTC
FUT2_E2_G2	AATCTTTGGCAGGTGAGCCC
CC2D2B_E45_G1	GACTTTTTATATTTACCTGTGAG
CC2D2B_E45_G2	GTTTTTCTATGAGCCAGAGG
CCT4_E11_G1	TGGGGTTTTTTTGACCCTTC
CCT4_E11_G2	CGAATAACACATAGGGCATC
PSKH1_E2_G1	ACACACCGAGACCTCAAACC
PSKH1_E2_G2	AATGTACTCAGGCGTGCCAC
AGL_E35_G1	TTTCCAGATTGATGGGCCCG
AGL_E35_G2	ACCTAGGGCATACAGAAATC
RAB40AL_G1	CGAGTCCCCGTACAGTCACC
RAB40AL_G2	TTGTGCACCACGAGAGTAGG
PRAMEF13_E4_G1	AGCTACGTGCTGCTGTTCCG
PRAMEF13_E4_G2	CAGGGCACCCATAGACATAC
GPR162_E2b_G1	TCATGGGTCTGGTCTGTGTG
GPR162_E2b_G2	AAGCTGGCCGGGTACCCAAG
SCN1A_E12_G1	TGGGGAAGAGAAAGATGAGG
SCN1A_E12_G2	CATGCATCAGTAAACTCAGC
RWDD2B_E4_G1	GGCTATGTCAGCAGAGATAC
RWDD2B_E4_G2	AGCTCCTTTGCCCACTCTAG

AKAP2_E13_G1	CAGGCTGTTCTCCATCAAGC
AKAP2_E13_G2	TCCCTTGGCGGCTGAGGCAC
SYNE3_E4_G1	GGAGCATGAGGAGTACCAGG
SYNE3_E4_G2	TTACCTGCAGTGTGGAGAGG
AKIRIN1_E3_G1	TTATAGGTGTGAACCACCGC
AKIRIN1_E3_G2	ATTTGCTCATACTCCTCCCG
TAS2R31_G1	TTTCCTCTTGTTATGTGCCG
TAS2R31_G2	CCTCACTTGCCGCAAAACTG
TRO_E12b_G1	GTCTTCAGTAGTGCGCTTAG
TRO_E12b_G2	GCTGGAAGAGCCACCAAAGG
CRTC1_E10_G1	ACCTGGCTCCTCTCCACAGC
CRTC1_E10_G2	TTTCCCTTTCCCCCTCCCAC
AKAP12_E5_G1	TCTGCACAGGATGAAACACC
AKAP12_E5_G2	GGAACCTTCTACCTCAACAG
CELA3B_E1_G1	GTGCCCTTTTCCTATCATCG
CELA3B_E1_G2	GATTTCAAGGATTCCTAATCT
NLRP8_E8_G1	ATTTTCCCCAGAACCATGGC
NLRP8_E8_G2	GTTCTCCAGGGAGTTTTTTC
NOVA1_E6_G1	AATTCGTACCTGGCACAAGG
NOVA1_E6_G2	CACTTTCTGAGGATTGGCAG
PHF8_E15_G1	GCTCTAACCCCCTCATGTAC
PHF8_E15_G2	AATGGGCTGGCCTGGTTAGG
RBM20_E9_G1	TTCACCTCCTGCAGCTCTTC
RBM20_E9_G2	TGTCATCTCCGTTGTCCCTC
KIAA0319L_E3_G1	CAGGGCTCTTTATGGAGCAG
KIAA0319L_E3_G2	TTTCTCCCCCAGATCTCAGG
COG5_E7_G1	GGCAACCCTGTTTAATAGCG
COG5_E7_G2	ACTTCAAGTCGGGCTCTTGC
SIPA1L2_E13_G1	CTGACGACGAGCCAGCCAAG
SIPA1L2_E13_G2	GGTACTACTCTGCAGTGCTG
AGAP3_E6_G1	ATCCCCGGGTTATCGACGAC
AGAP3_E6_G2	AGTGACTCCCACCCGGCACC
UBE2Q1_E5_G1	AGAAGACTTAGATCACTATG
UBE2Q1_E5_G2	AGTAATCTTGCCTCTGGTTC
SAMSN1_E4_G1	ATACTTATCAGTTTGGACAGTG
SAMSN1_E4_G2	TGGAATAAACTACGAACCTC
ZNF175_E5_G1	CACAACTCAAGGTGCATCAG
ZNF175_E5_G2	CTCTGGTATGAGTTGTTTGG
MCHR1_E1_G1	CTTGCGCTCCGGGACAAGG
MCHR1_E1_G2	GCAGCAGCGAGGCTTCCAGG
NPAP1_E1_G1	CTCCTGCTTGGAAGGCCCTG
NPAP1_E1_G2	TCCGCAGCCAACTTCTGCTG
FRMD1_E3_G1	GTGCCGGCCCCTCCCTGAAC
FRMD1_E3_G2	TGACCACACAGAGGCCAAAG
CKMT2_E8_G1	GATAAGACATTTCTCATCTGG
CKMT2_E8_G2	CTTTCAAGAACAAACAATACGG
TYRO3_E2_G1	GCTGACACATGTTCCTTCCC
TYRO3_E2_G2	GCTGGCAGCCCACACCTTC
PSIP1_E4_G1	TTTGCACTCACATCTTCCCC
PSIP1_E4_G2	GATAGTGATTATTCCCCAGG
PPP45R1_E14_G1	ACTCTTCACTATATTCACAGCG
PPP45R1_E14_G2	GGAATTAGGAACACACCCAC
RBKS_E7_G1	AATCATTACCTTAGGGGCTG
RBKS_E7_G2	TTTCAAAGGCTAAACATGATTC
WFI1_E7_G1	TGCTTTAATGGAGGGACCTG
WFI1_E7_G2	ATGGGGTTTCTAAGGTCTCC
SAT2_E2_G1	CCCTTCTGCTCTAATCTAGC
SAT2_E2_G2	AGCCTCCGCCAGAACCTGGG
NFE2L3_E4_G1	ACTTACCACTTACAGCCAAC
NFE2L3_E4_G2	TGCAAAGCTTTAGCACGCTG
MGAM_E10_G1	TTCTTTCAGCTCATTGGGCG
MGAM_E10_G2	TACATAAGGGAGCTGTGCTG
FAT4_E3_G5	GCTAGTTCGTGCTGATGATG
FAT4_E3_G6	CACATAAGAGACCCATCACC
FAT4_E17_GS1	CAAGCAGGGACTTTCAACTG
FAT4_E17_GS2	GTCCCAGCTTTGCCTTCTTC
FAT3_E20_G1	ACTTCTCTCAGCTTTGCTGG
FAT3_E20_G2	AGGGATTTGCTCTGGTGTAC
PHTF2_E6_G1	CCCTTTTTCTTCCGGTGGTG
PHTF2_E6_G2	CCTAACAATGTAACATTCTG
CHRM1_E2_G1	AGGAGCCTGGCTCCGAAGTG
CHRM1_E2_G2	GCCCTTTCTTAGTCGGCCTC
BCR_E8_G1	TGCAGGACGCCCTCCGCATC
BCR_E8_G2	CAAGCCATCCCCTGCCACAC
RNF111_E9_G1	GGTGCCTATGTAACATACTC
RNF111_E9_G2	TAATCCACTTGAGGCGGAGG
AHNAK2_E7_G1	GCATTCAGCCTGCTTCTACT
AHNAK2_E7_G2	CTTCCGCCTTGGGGCTTTTC
TOP1MT_E8_G1	ATAACACCGTCACGTGGCTG
TOP1MT_E8_G2	ACCTCCTGGGGAGGAAACAC
BRCA2_E18_G1	GACTGACAGTTGGTCAGAAG
BRCA2_E18_G2	CTGATTTTTACCAAGAGTGC
PABPC1_E8_G1	CTCCTCATTCTACCATTACG
PABPC1_E8_G2	TTCTGTAACTGCTTTAGTGG
ABCC6_E24_G1	ACGTTCCAGGGCAGCACAGT
ABCC6_E24_G2	GCTCTTCCTACCTGTCAGCC
FAT1_E11_G1	TACCTTCTTACTATCACGGC
FAT1_E11_G2	CACAAATGTAAGGGAAGAGC
PER3_E17_G1	ACCCCCTTCAGTCACCAGCC
PER3_E17_G2	GGTCTGGGCATCTCTTCCTG
CACNB2_E19_G1	ACACCACAACCATCGCAGTG
CACNB2_E19_G2	GGGAATAATCTTCCTTTGGC
GBF1_E11a_G1	TGGAGTCCATCCCTGAAGTG
GBF1_E11a_G2	CCCACCTTCTTTCTGGGAGG
GSE1_E16_G1	CTTCGGGCTCCTGCATACGG
GSE1_E16_G2	GCTGTGGGAGAGCTGCTCTG
ZNF287_E6_G1	GGTAAAACCTTCACCCAGAG

ZNF287_E6_G2	CTGAATGTTCTGACACATAG
CCDC102B_E5_G1	CTGCACTGCCAACTGGAGAG
CCDC102B_E5_G2	TTCTGAGGTGGCAAACTCTG
SPRY1_E5_G1	GATCCCCAAAATCAACATGG
SPRY1_E5_G2	CTTATGGCCTTGATCTGGTC
HHIP_E5_G1	TTCAGGAGGTTGTGAGTGGG
HHIP_E5_G2	CTCCTTCAGGGGTAAGTATC
ELL2_E8_G1	TTTAGTGCAAACTGGCTCAC
ELL2_E8_G2	CCATTTAGTGTTGGTGGTAC
TMEM130_E6_G1	AAGGGGAGTGCCACCCTGTG
TMEM130_E6_G2	GGTACTGATGTGTCTTGCTG
ZNF655_E2_G1	CCTGGAGAGAGAGTTTAGGC
ZNF655_E2_G2	TCATTGTCATCGGTATTCTG
VEPH_E7_G1	GCTGTGTATGAAAAGCAGCC
VEPH_E7_G2	GCTGCTACATGCAAAAGCCG
SLC35G2_E2_G1	AAGAAATGGATACTTCTCCC
SLC35G2_E2_G2	TCTTCATATCCATCATCGCC
KIF9_E5_G1	TTTTCCTGGATGTCACCGTG
KIF9_E5_G2	TCCCCCGGTGCTTGTAATTC
WDR6_E2_G1	AGGTGGCCCTCAGGATCCTC
WDR6_E2_G2	ATGACATGGCAGGCGAGGCG
CCDC171_E24_G1	CCCTTTAGGTCAGAGATCAG
CCDC171_E24_G2	CTAACCTGGCATGCTACCAC
FAT4_E1_G3	TGCTTCTGACAGAGCAGTGG
FAT4_E1_G4	ACTCAAAATCTGGCTGCACC
ABCC4_G1	GTCTACTTTTTGCATCTTTACG
ABCC4_G2	ACCATTATCTTGTCGCTGTC
ABCC4_G3	AATTGGCCCCAAGTCACGG
ABCC4_G4	CCTTTTTCAGAGCACAAGCC
ABCA4_G1	ATGTCCTTCGTCCCAGCCAG
ABCA4_G2	GAGGCGCTGTAAACTGACAC
EHD2_G1	GACCAGCTTCATCCAGTACC
EHD2_G2	TTGTCCGGGTCCACGACGAG
AHNAK2_G3	ACTGACCTCAGCGTCCAGAC
AHNAK2_G4	TCAAACTGGGCCTCTGCACC
THBS2_G1	ACAACACTGACCAGAGGGAC
THBS2_G2	CTCTGATGAGGACCTCCAAC
ZFHX3_G1	CCAAAGAGGCCAATCGGAAG
ZFHX3_G2	ATCAGGGCAGCCTGTTGCTG
ZFHX3_G3	ACAGTACAGAGACCACTACG
ZFHX3_G4	AGCTCCATCGGCATGGAGAG
FAT4_G_101	CAACCCACCTGTATTCAGTC
FAT4_G_102	GGAATTGAGATCCATGGTGG
FAT4_G_103	ACGGGCCCATGCTGACTGTC
FAT4_G_104	GGACCTTGATTTGGAGGGAG
FAT4_G_105	GAGATATTATCCACCCTACTC
FAT4_G_106	CATTTACCTGTTTGGGCATC
FAT1_G_101	CACTTTCATCCACAGACACC
FAT1_G_102	CCGTGACTGACATTGAGGAAATC
FAT1_G_103	GGGCAGTCCACCAATGAGTG
FAT1_G_104	CCCAATGCTGACAGTTTCAC
FAT1_G_105	ATGGTAAAGGCCATTCCTGC
FAT1_G_106	GCCTGCTGTCTTTTAACTGG
FAT1_G_107	GTGAAGGATCACTACACATTG
FAT1_G_108	TGGGTGAGAATAACGGTCTC
FAT1_G_109	GCTCCTGCTTCTGCTCCTTC
FAT1_G_110	AGTTCTCCTGCACGGTGACG
BRCA1_G_17	CCATCTTCAACCTCTGCATTG
BRCA1_G_18	GCTCACACTTTCTTCCATTGC
BRCA1_G_19	TGACCTGTTAGATGATGGTG
BRCA1_G_20	CCCTGAGCCAAATGTGTATG
BRCA1_G_21	ATTCCAAGTACAGTGAGCAC
BRCA1_G_22	GGAGCCCACTTCATTAGTAC
BRCA1_G_23	GGATTTATCTGCTCTTCGCG
BRCA1_G_24	GGAGATAATCATAGGAATCCC
BRCA2_G101	GATAACAAATATACTGCTGCCAG
BRCA2_G102	TGATCAGTAAATAGCAAGTCCG
